# Effects of Using Human Patient Simulator (HPS™) versus a CD-ROM on Cognition and Critical Thinking

**DOI:** 10.3885/meo.2008.T0000118

**Published:** 2008-01-30

**Authors:** Don Johnson, Amanda Flagg, Theresa L. Dremsa

**Affiliations:** * US Army Graduate Program in Anesthesia Nursing, Fort Sam Houston, San Antonio, Texas USA; † School of Nursing, University of Texas Health Science Center, San Antonio, Texas USA; ‡ United States Air Force Retired, San Antonio, Texas USA

**Keywords:** simulation, teaching strategies, cognition, critical thinking

## Abstract

**Background:**

Very little prospective randomized experimental research exists on the use of simulation as a teaching method, and no studies have compared the two strategies of using the HPS™ and a CD-ROM. In addition, no researchers have investigated the effects of simulation on various levels of cognition, specifically lower-level and higher-level cognition or critical thinking.

**Objectives:**

A prospective pretest-posttest experimental mixed design (within and between) was used to determine if there were statistically significant differences in HPS™ and CD-ROM educational strategies in lower-level, higher-level cognition and critical thinking.

**Results:**

A repeated measures multivariate analysis of variance (RMANOVA) with LSD post-hoc tests were used to analyze the data. There were no significant differences between the HPS™ and CD-ROM groups on lower-level cognition scores. The HPS™ group did significantly better than the CD-ROM group on higher-level cognition and critical thinking scores.

**Conclusion:**

This study demonstrated that the choice of teaching strategies for lower-level cognition does not make a statistically significant difference in outcome. However, the HPS™ is superior to using CD-ROM and should be considered as the choice in teaching.

Never has chemical warfare presented such a real threat as it does today. In situations where individuals are exposed to chemical warfare such as in combat or terrorist attacks, healthcare personnel are among the first responders. Therefore, these professionals must have the necessary cognitive and critical thinking skills to manage patients exposed to chemical agents. The purpose of this study was to evaluate whether the Human Patient Simulator (HPS™) (Medical Education Technologies, 2005) or an interactive CD-ROM was more effective in increasing cognition and critical thinking relative to the care of combat casualties exposed to chemical agents.

## Background

**Human Patient Simulation** - Simulation has been part of healthcare education since the 1950's when nursing students practiced on Mrs. Chase, a static doll-like mannequin.^1^ Simulation is defined as a realistic representation (model) of the dynamics or processes with which the participant interacts with the environment, applies previously learned knowledge into the decision making process, and responds with definitive decisions and actions to deal with a problem or situation. Performance feedback is provided without concern regarding real-life consequences.^2^ This type of simulation can be accomplished through the use of the HPS™ or an interactive CD-ROM as used in this study.

Simulation has advanced far beyond Mrs. Chase to a very interactive, talking model available today. The HPS™ was developed through the cooperation of healthcare professionals and computer technology. The HPS™ is a computerized full-body mannequin that is capable of providing real-time physiological and pharmacological responses to various health condition and pharmacological interventions. The complete HPS™ system includes the mannequin, computer software, monitors and gases required to operate the system. A cordless microphone located in the mannequin's head is used to simulate the “patient's voice.” Participants are able to ask the mannequin questions, and the operator is able to respond by transmitting his/her voice through the mannequin. Observation is possible from a separate room through a closed circuit television monitor. Participants can assess all physiologic parameters including normal and abnormal heart and lungs sounds. They can also palpate pulses, check pupil response, obtain vital signs, and monitor rhythms, pulse oximetry, blood pressure, and respiratory rates.

In this study, the HPS™ was programmed with three patient scenarios: exposure to a nerve agent with an abdominal wound and subsequent hypovolemic shock, exposure to a nerve agent only, and exposure to a mustard gas. The HPS™ was programmed to manifest signs and symptoms relative to nerve agent exposure that included pin-point pupils, copious secretions, lung crackles, rhonchi, perspiration, blood pressure and pulse changes. Participants were able to obtain a manual blood pressure and palpate pulses. The HPS™ was attached to a cardiac monitor, Life Pak 12 Defibrillator, so that blood pressure, pulse, and cardiac rhythms could be assessed by observing the monitor. In the hypovolemic scenario, the monitor demonstrated that the patient had hypotension with tachycardia. In this scenario, radial and brachial pulses were absent but carotid pulses present. HPS™ provided participants with the ability to assess, make a diagnosis, intervene and evaluate the intervention. Appropriate physiological responses to pharmacological interventions such as administration of fluids or atropine were immediately demonstrated by the HPS.™ For example, in the hypovolemic model, fluid administration resulted in a decrease in pulse and an increase in blood pressure. After an adequate amount of fluid was administered, radial and brachial pulses were palpable. The administration of oxygen resulted in increased saturation as monitored by pulse oximetry. The administration of atropine resulted in a reduction in secretions, crackles, rhonchi, perspiration, and an increase in pupil size. The HPS™ allowed programming of two complex problems to be manifested simultaneously in the same patient scenario: exposure to a chemical agent and hypovolemic shock.

**Chemical Warfare CD-ROM** - A CD-ROM was developed for this study that allowed participants to view PowerPoint slides covering the pathophysiology of chemical agent exposure and hypovolemic shock. In addition, the same three scenarios developed for the HPS™ were presented on the CD. With each scenario, the participant was able to assess each patient by clicking a selection of choices. The choices included assessment parameters such as vital signs, auscultation of the chest, and visual inspection of an abdominal wound as examples. After the choice was made, the CD-ROM provided the appropriate information that was queried. The participant made a diagnosis by clicking on a selection of choices. The participants were also able to click on various treatment options such as administration of oxygen, intravenous fluids, and other treatment modalities needed to stabilize the patient. After completion of each of the scenarios, the CD-ROM gave feedback on each decision and evaluated the participant's performance. Each of the scenarios for both the HPS™ and CD-ROM groups took approximately 30 minutes each to complete for a total of 90 minutes for the three scenarios.

**Simulation** - Previously, research has not used the HPS™ or the CD-ROM strategy for teaching care of patients exposed to chemical agents. Very little prospective randomized experimental research exists on the use of simulation as a teaching method, and no studies have compared the two strategies. In addition, no researchers have investigated the effects of simulation on various levels of cognition, specifically lower-level and higher-level cognition or critical thinking. No studies address the score reliability and validity of the instruments used in the assessment of participants. A wealth of literature addresses the value of using simulation as a teaching method but fails to use a rigorous research design.^3^–^10^ For example, McIndoe surveyed participants and found that the majority preferred problem-based simulation to lecture, rounds, or tutorial teaching formats, but he did not investigate the effectiveness of such an approach.^11^ Rauen found that simulation as a method of teaching allows learners to apply theory to practice in an integrated manner. Furthermore, she found that simulation demonstrates more than a single event or parameter at a time which allows participants to identify relationships essential and common to clinical practice. She found that the evaluation of the simulation sessions was universally positive. As a result of the use of simulation, students became confident and were able to demonstrate skills learned.^12^ However, Rauen did not compare the simulation approach to any other method or to a control group. Gordon, et al., surveyed both students and educators about their opinions about simulation as a teaching tool. Both groups thought that the advantage of using the stimulator outweighed the disadvantage of the cost of the simulator.^13^ Eaves and Flagg created a 10-bed simulated medical unit as part of a new graduate nurse orientation. The program received outstanding evaluations from the new graduates, the educators, and preceptors in the clinical setting where the new graduates practiced. The study did not compare the simulation with any other methods.^14^ Recently, Cioffi, et al. investigated the effectiveness of simulation on clinical decision making by midwifery students; however, the study used a posttest design with no mention of score reliability or instrument validity.^15^ Results showed that the students who received the simulation strategy collected more clinical information, had higher confidence levels, and reached a final decision more quickly than the lecture group. Furthermore, investigators advocated the need for rigorously designed pretest/posttest studies of simulation that would include a comparison group.^16^

**Framework for Study** - The framework for this study is the integration of Bloom's taxonomy and critical thinking. Bloom recognized that there are different levels of cognitive skills or thinking, which he classified as the cognitive domain (See Table 1).^17^

**Table 1:** Summary of Bloom's Taxonomy

Cognitive Level	Description
Knowledge (Remembering)	Ability to remember facts. This represents the lowest level of learning outcomes in the cognitive domain.
Comprehension (Understanding)	Ability to grasp the meaning of information by translating material from one form to another
Application (Applying)	Ability to use the material in a new situation and includes the use of principles, concepts, laws, and theory.
Analysis (Analyzing)	Ability to break down material into its component parts. There may be identification of the parts, examination of the relationships between parts, and recognition of the organization principles involved.
Synthesis (Evaluating) Ability to put parts together to form a new whole	Ability to compose, design, formulate and plan
Creation (Evaluation)	Ability to judge the value of material or act for a given purpose. The individual can appraise, judge, revise and/or value. This implies that there are criteria, which may be developed by the learner or given to the learner by the instructor.

The investigators theorized that lower-level cognitive skills of knowledge and comprehension are necessary for higher-level skills of application through creation. In addition, the researchers theorized that these higher-level skills are necessary for critical thinking.

**Critical Thinking** - Critical thinking has its origins in the Socratic reasoning characterized by combining abstract thinking and logical thinking that requires rational and objective processes involving order, structure and sequence. Today, critical thinking is defined in a variety of ways.^18^ May summarized definitions from various authorities and concluded that critical thinking is a process, a composite of knowledge, attitudes and application skills with cognitive skills and dispositions.^19^ Furthermore, she reasoned that such thinking involves the examination of ideas, inferences, assumptions, principles, arguments, conclusions, issues, statements, beliefs, attitudes, and actions. These components serve as a means for making decisions regarding practice and providing care. Consensus among an international panel emphasized the importance of critical thinking as an essential component of professional accountability and quality health care.^20^ Other theoretical views suggest it as a process of evaluating the many complexities of a situation in order to determine what is meaningful and relevant.^21^,^22^ Rauen summarizes critical thinking as a process of reflective thinking that goes beyond logical reasoning to evaluate the rationality and justification for actions within the context of a situation. The focus is not only on figuring out answers but on achievement of an understanding within the context of a situation.^23^

Debate continues about the nature of critical thinking, the transferability of critical-thinking skills from one setting or situation to another, and how to teach these skills. Some authors describe critical thinking as a generalized skill with applicability to a variety of situations and contexts.^24^ Others claim critical thinking must be developed within the context of the subject matter and discipline.^25^ Alfaro-LeFerve asserted that critical thinking is contextual, requiring job specific knowledge, and must be mastered within that context.^26^ Facione stated that critical thinking is a purposive, self-regulatory judgment and is a construct that greatly overlaps the boundaries of clinical judgment. Critical thinking is subject-matter specific, requiring skills inherent within the diagnostic and treatment thinking process. These skills include analyzing, applying standards, discriminating, information seeking, logical reasoning, predicting, and transforming knowledge as they relate to the management of chemical warfare casualties.^27^ Healthcare decision making incorporates assessment, data recognition, and planning that includes goal setting, priority setting, and selection of intervention measures. Implementation and evaluation of these components represent critical thinking relative to specific content areas.

Critical thinking in the care of chemical warfare casualties requires all the skills mentioned. The healthcare providers are required to seek information, collect data, discriminate between relevant and nonrelevant data, analyze situations, apply standards of care, use logical reasoning, and perform the appropriate skills. Healthcare providers must possess the cognitive skills and critical thinking ability to care for patients exposed to chemical agents.

**Operational Definitions** - For the purposes of this study, the following operational definitions were adopted:

1. Lower-level cognition represents the knowledge and comprehension level using Bloom's (1956) taxonomy relative to the pathophysiology of chemical warfare and care of patients exposed to chemical warfare.^28^ This is defined as the score ranging from 0 to 100% on the Lower-Level Cognition Instrument.
2. Higher-level cognition represents application, analysis, synthesis and evaluation relative to the pathophysiology of chemical warfare and care of patients exposed to chemical warfare. This is defined as the score ranging from 0 to 100% on the Higher-Level Cognition Instrument.
3. Critical thinking represents skills of assessment, intervention, and evaluation inherent in diagnosis and treatment processes. This is defined as the score ranging from 0 to 100% on the Critical Thinking Instrument.

**Problem Statement** - It is not known which of two teaching strategies, the use of a Human Patient Simulator (HPS™) or an interactive CD-ROM, is more effective in increasing cognition and critical thinking relative to care of casualties exposed to chemical agents.

**Research Question** - The research question guiding the study was as follows: Is there a statistically significant difference in HPS™ and CD-ROM educational strategies reflected in scores on the Lower-Level Cognition, Higher-Level Cognition, and Critical Thinking Instruments?

## Methods

A prospective pretest-posttest experimental mixed design (within and between) was used to determine if there were statistically significant differences in HPS™ and CD-ROM educational strategies. Participants were healthcare providers recruited from various active duty and reserve US Army and US Air Force units in south Texas. Potential participants were told the purpose of the study and assured that participation was voluntary. Individuals who chose to participate in the study completed the instruments and then were randomly assigned to one of three groups: use of HPS,™ use of the CD-ROM, or the control group. The participants in the HPS™ and CD-ROM groups then received instruction as described above. One month after the pretest data were collected and participants took part in one of the three groups, they then returned and completed the same instruments. Participants in the control group received no instruction but were given the opportunity to complete the HPS™ educational teaching strategy after all the data were collected. The study was approved by the local Institutional Review Board (IRB).

**Setting** - The study was conducted at a large military medical center located in the southwestern part of the United States. The Air Force War Skills Simulation Laboratory at a large southwestern medical center has been in operation since September 2000. The simulation laboratory is a fixed facility configured to represent a typical wartime medical care environment. For example, the walls are covered with camouflage netting and reinforced with sandbags. Bruce lights (field lights) furnish the lighting. Equipment included field gear used to transport equipment and supplies and only the equipment available in a deployed environment such as a PT LOX (equipment used to produce field oxygen), field ventilator, Lifepak 12 Defibrillator, and a transport intravenous (IV) pump. The simulation laboratory was also equipped with video recording equipment and a stereo sound system to allow for further recreation of a battlefield environment. The centerpiece of the simulation laboratory is the HPS.™

**Sample** - The convenience sample used for this study consisted of 99 active duty and reserve healthcare volunteers who completed both the pretest and posttest instruments. Eight individuals did not return to complete the posttests and were excluded from the study.

**Cognitive Performance Instrument** - Learning objectives were developed for three chemical warfare scenarios based on Bloom's taxonomy. The objectives represented lower-level and higher-level cognition and critical thinking skills related to care of casualties exposed to chemical agents. A test blueprint was developed to guide the item writers in the development of a total of 90 multiple-choice questions. The multiple-choice questions were written to represent lower-level and higher-level of questions and critical thinking. Before developing questions, item writers participated in a workshop on the development of psychometrically valid items.

**Content Validity** - Validity is defined as the degree to which the instruments measured what they purported to measure. Content validity is concerned with adequately sampling the content material and determining if the Lower-Level, Higher-Level and Critical Thinking Instruments are representative and comprehensive. To develop content validity, the investigators reviewed the literature on chemical warfare to determine content that needed to be taught and tested for each of the scenarios. Polit and Beck state that establishing content validity is aided by consensus in the literature.^29^ After review of the literature, the investigators developed objectives for the cognitive instruments based on Bloom's taxonomy for lower (knowledge/comprehension) and higher (application, analysis, synthesis, evaluation) cognition levels. The objectives were further developed into critical thinking objectives reflecting assessment, intervention, and evaluation domains. Several objectives overlapped, as application objectives reflected critical thinking objectives and vice-versa; therefore, these objectives were dual-classified. A test blueprint was constructed to represent the number of items needed in each area. Items were then developed that reflected the test blueprint. The items were given to six experts in education and chemical warfare consisting of two critical care nurses, one emergency department nurse, two trauma physicians, and one chemical warfare educator. The experts individually rated each question as very pertinent, pertinent, not pertinent, or not at all pertinent. Items rated as not pertinent were excluded from the instruments. Items were also excluded if the experts could not agree on the classification level or the correct answer. Questions were categorized as lower level, higher level, or critical thinking. Sixty-six questions remained, representing 100% content validity by the standards described.

**Item-Objective Congruence** - The focus of item validity was to determine how well each item in the instruments represented the specific objectives. The expert panel was provided the question and the objective that the investigators believed represented the individual item. The experts were asked to evaluate each item and determine if it was an appropriate measure of the content domain specified in the test blueprint. A value of +1, 0, or –1 was assigned for each item, reflecting the congruence of each item with the objective. When an item was judged to be a definite measure of the objective, a value of +1 was assigned. A rating of 0 indicated that the judge was undecided about whether the item was a measure of the objective. The assignment of –1 rating reflected a definite judgment that the item was not a measure of the objective. Hence, the task of the content experts was to make a judgment about whether or not an item fell within the content domain as specified by the objective of the instruments. The limits of the index could range from -1.00 to a +1.00. Items scored less than +1.00 were deleted so that the final instruments had an index of +1.00, indicating excellent positive item-objective congruence.^30^

**Determination of Reliability** - Reliability refers to a property of test scores. Specifically, consistency or repeatability of the scores on an instrument can be conceptualized in terms of stability. To determine the stability of the measures, the investigators used a test-retest procedure for lower-level cognition, higher-level cognition, and critical-thinking questions. The instruments were administered to 30 healthcare providers and readministered under the same conditions to the same group one month after the first data collection, as recommended by Jacobson.^31^ Participants were not exposed to relevant content during this one-month interval. Those who had any training or classes relative to chemical warfare were excluded from the study. Scores were computed on the Lower-Level, Higher-Level, and Critical Thinking Instruments. Scores on the first observation were correlated with the second observation using the Pearson product-moment coefficient. A correlation coefficient of 0.80 was obtained, indicating an acceptable test-retest correlation and acceptable test score reliability for a new instrument.^32^ After excluding questions on which the expert panel disagreed, the investigators used the Pearson product-moment coefficient and found a correlation of +1.00, indicating a perfect test-retest reliability. The Kuder-Richardson Formula 20 (KR-20) is a measure of internal consistency reliability for measures with dichotomous choices and is analogous to Cronbach's α, except Cronbach's α is used for non-dichotomous (continuous) measures. The KR-20 coefficients for the pretest and posttest were .88 and .92, respectively indicating that the instrument had an acceptable degree of internal consistency.

**Readability of Instrument** - To make sure that the items were written at a level that the participants could comprehend, the investigators evaluated the readability of the instruments. The Flesch Reading Ease formula^29^ rates text on a 100-point scale: the higher the score the easier the text is to understand. A target range of 60-70 is desirable for most documents. The score for the instruments was 60 indicating they were fairly easy to read. Another method of determining readability is the use of the Flesh-Kincaid Grade level which rates text on a United States (US) grade-school level.^29^ A score of 8 means that an eighth grader could understand the document. The score is based on the average sentence length and the average number of syllables per word. Although the Flesch-Kincaid Grade Level score for the instruments was 14, the investigators considered this acceptable because all of the participants had at least a baccalaureate degree.^33^

Power Analysis-The number of participants needed for this study was calculated using an alpha of 0.05, moderate effect size of 0.5, and power of 80% which yielded 90 participants (30 in each group).^34^ The medium effect size was estimated from data from a pilot study implemented by the investigators.

## Results

A multivariate analysis of variance (MANOVA) was used to determine if there were significant differences in the pretest scores between the groups (HPS,™ CD-ROM, and control groups). A repeated-measures multivariate analysis of variance (RMANOVA) and LSD post-hoc analyses were used to determine if there were significant differences between the groups over time. An alpha of 0.05 was used for all analyses. The assumptions of the RMANOVA include that the dependent variables are measured on an interval or ratio scale, there is homogeneity of variance, and data are normally distributed. The scores on the dependent variables were measured on a scale of 0 to 100 percent and were therefore considered ratio data. Levene's test was used to determine if the assumption of homogeneity of variance was met. For each variable, Lower-Level Cognition, Higher-Level Cognition, and Critical Thinking Instrument scores, the assumption was met (p = 0.41, 0.42, and 0.42, respectively). The assumption of the data's normality was evaluated by the skewness of the scores. The skewness for each dependent variables was as follows: Lower-Level Cognition Instrument Scores =.12; Higher-Level Cognition Instrument Scores = -.23; Critical Thinking Instrument Scores=.12. The standard error for the scores on each instrument was 0.253. A skewness value more than twice its standard error indicates a departure from symmetry. Since there was no departure greater than this value, the assumption of normality was met.

The results from the MANOVA indicated that no significant differences between the groups on the pretest scores on the Lower-Level Cognition (p=.98) Higher Level Cognition (p=.57) or the Critical Thinking Instruments (p=.68). Hence, the groups were considered equivalent on all of the pretest (baseline) scores. The Wilks’ Lambda multivariate test indicated that there were significant differences in group means by time (p = 0.00). The LSD post-hoc indicated that there were significant differences between the groups on Lower-Level Cognition scores (p = 0.001). However, there were no significant differences between the HPS™ and CD-ROM groups (p = 0.143) and the CD-ROM and control groups (p = 0.333). There was, however, a significant difference between the HPS™ and control groups (p = 0.017) See Table 2 for a summary. A significant effect was found between the groups on Higher-Level Cognition scores (p = 0.001). Post-hoc analysis indicated significant differences between the HPS™ and CD-ROM groups (p = 0.021) and the HPS™ and control groups (p = 0.011). There was no significant difference between the CD-ROM and control groups (p = 0.80). A significant effect was found between the groups on the Critical Thinking scores (p = 0.001). Post-hoc analysis indicated a significant difference between the HPS™ and CD-ROM groups (p = 0.038) and the HPS™ and control groups (p = 0.010). There was no significant difference between the CD-ROM and control groups (p = 0.603).

**Table 2:** Summary of Results

Instruments	Mean Test Scores (±sd) Human Patient Simulator (HPS^TM^) Group	Mean Test Scores (± sd) CD-ROM Group	Mean Test Scores (± sd) Control Group	Post-Hoc Analyses		
Lower-Level Cognition Pretest	53.39±11.97	52.70±13.54	53.07±10.8	HPS™ vs. CD p = 0.143	HPS™ vs. control p = 0.017*	CD vs. control p = 0.333
Lower-Level Cognition Posttest	70.85±10.61	64.09±9.52	59.00±10.37			
Higher-Level Cognition Pretest	60.56±9.20	57.46±12.77	58.67±11.73	HPS™ vs. CD p = 0.021*	HPS™ vs. control p = 0.011*	CD vs. control p = 0.80
Higher-Level Cognition Posttest	73.92±9.08	65.22±9.97	62.80±10.98			
Critical Thinking Pretest	62.41±11.19	59.67±13.67	60.43±12.34	HPS™ vs. CD P=.038*	HPS™ vs. control P=.010*	CD vs. control p=.603
Critical Thinking Posttest	77.01±8.74	68.70±11.50	65.27±10.49			

*mean difference is significant at the 0.05 level

## Discussion

The results of this study indicate that the choice of teaching strategies for lower-level cognitive tasks does not make a statistically significant difference in outcome. The data from the study suggest that there is no statistically significant difference in the use of the HPS™ compared to a CD-ROM for increasing lower-level cognition relative to caring for chemical warfare patients. This finding may be related to the fact that knowledge is a function of remembering facts, and comprehension is the ability to grasp the meaning of information. These levels of cognition represent the lowest level of learning outcomes. Although there were no statistically significant differences in the scores on the Lower-Level Cognition Instrument between the HPS™ and the CD-ROM groups, the HPS™ group scored 70.85% compared to the CD-ROM group that scored 64.09%.

The investigators found that the use of HPS™ compared to a CD-ROM resulted in a statistically significant difference in outcome for higher-level cognition and for critical thinking. The findings may be related to the fact that teaching strategies using the HPS™ provide the opportunity for learners to apply principles, concepts, laws, and theory more than other strategies. The HPS™ allowed participants to use the cognitive skill of evaluation and treatment in a realistic simulated environment. Specifically, in this study participants were able to evaluate the effectiveness of atropine for nerve agent exposure. For these scenarios, HPS™ demonstrated an increase in pulse, dilation of pupils, and a decreased presence of rhonchi. For the scenario using hypovolemic shock, participants were able to evaluate the effectiveness of fluid resuscitation as evidenced by an increase in blood pressure and decrease in pulse. Such skills represent the higher-cognitive and critical-thinking skills of assessment, intervention, and evaluation. The effectiveness of such a strategy stems from the theory of situated cognition that states that individuals best learn “what to do” and “how to do” in a real world environment.^35^ Accordingly, situated cognition asserts that critical thinking has to occur within the context of the situation. Knowing what to do and knowing how to do it are essential components of higher-level cognition and critical thinking in the care of chemical warfare casualties. This concept has to be developed within the specific subject matter and is best taught under realistic simulated conditions that best represent the desired patient-care conditions.

This was the first study to investigate cognitive scores representing lower-level cognition, higher-level cognition and critical thinking to compare the effectiveness of HPS™ and CD-ROM teaching strategies. The study used a pretest/posttest 3-group prospective randomized design. Pretest scores of the control group, CD-ROM group, and HPS™ group did not differ, indicating that the groups had approximately the same initial scores on the instruments. The posttest scores indicated a significant difference between the HPS™ and control groups and the HPS™ and CD-ROM groups, but there was no statistically difference between the CD-ROM and control groups. The results strongly suggest the use of a HPS™ is very effective in teaching higher-level cognition and critical thinking skills relative to caring for patients exposed to chemical agents.

Limitations of the study include the use of a convenience sample, which may restrict the generalizability of findings. The investigators assessed only face and content validity. Future research could use factor analysis techniques to examine the structure of scale scores. Additional recommendations for future research include using the same framework for different content and investigating the effects of simulation on actual performance.
